# Protective effects of *Delonix regia* and gum Arabic against aluminum chloride-induced toxicity in male Albino Rats

**DOI:** 10.14202/vetworld.2024.2909-2917

**Published:** 2024-12-19

**Authors:** Amin A. Al-Doaiss, Mohammed A. Alshehri, Ali A. Shati, Ali M. Almaawari, Hamad S. Al-Shahrani, Abdulaziz M. Saeed, Abdulaziz M. Al-Ghamdi, Abdulaziz S. Al-Shahrani, Muteb H. Almansour, Ahmed A. El-Mansi, Mohammed Al-Zharani, Mohammed Mubarak, Montaser Elsayed Ali

**Affiliations:** 1 Central Labs, King Khalid University, AlQura’a, Abha, P.O. Box 960, Saudi Arabia; 2 Department of Biology, College of Science, King Khalid University, P.O. Box 9004, Abha, 61413, Saudi Arabia; 3 Department of Biology, College of Science, Imam Mohammad Ibn Saud Islamic University, Riyadh, Saudi Arabia; 4 Department of Animal Productions, Faculty of Agriculture, Al-Azhar University, 71524, Assiut, Egypt

**Keywords:** aluminum, *Delonix regia*, gum Arabic, toxicity, Wistar Rats

## Abstract

**Background and Aim:**

Aluminum (AL) is commonly found in food, drinking, air, and soil and it can be a serious contaminant in varying amounts. Therefore, this study investigated the biochemical and histological hazardous reactions to aluminum chloride (AlCl_3_) and the efficiency of the methanol extract of *Delonix regia aerial* parts with gum Arabic (*GA*) as anti-toxic therapies to return to a natural state after AlCl_3_ exposure.

**Materials and Methods:**

A total of 20 male Wistar rats were randomly divided into four equal groups. (i) CG: Served as a control group. (ii) AlCl_3_: Rats were exposed to 80 mg/kg/body weight (BW) AlCl_3_. (iii) AlCl_3_ + *D. regia/GA*: rats were administered AlCl_3_ + 100 mg/kg B.W. with 15% BW of *D. regia* and GA, respectively. (iv) *D. regia/GA*: Rats were administered 100 mg/kg B.W. with 15% BW *D. regia* and *GA*, respectively. The experimental treatment was administered for 30 days. On the 30^th^ day, blood biochemical parameters were assessed, and specimens from the liver and kidney were collected and stored in a neutral buffer with 10% formalin until immediate histopathological examination after euthanasia.

**Results:**

This study revealed a significant increase in white blood cells and platelets after AlCl_3_ exposure compared with CG, while there was a decrease in red blood cells, hemoglobin, hematocrit, and mean corpuscular volume. Treatment with *D. regia/GA* improved lymphocytes, monocytes, eosinophils, and basophils. Furthermore, the animals exposed to AlCl_3_ showed a significant increase in aspartate aminotransferase, alanine aminotransferase, and alkaline phosphatase compared with CG, whereas AlCl_3_ + *D. regia/GA* treatment improved these activities. In addition, the rats exposed to AlCl_3_ had significantly increased glucose, lipase, amylase, triglyceride, cholesterol, high-density lipoprotein, and low-density lipoprotein levels, and *D. regia/GA* treatment significantly improved these levels compared with AlCl_3_. This study reported no significant differences in Ca and Na concentrations among groups, but rats exposed to AlCl_3_ had elevated K, Cl, and Mg levels, whereas *D. regia/GA* treatment improved these levels.

**Conclusion:**

The co-administration of the methanolic extract of *D. regia* with *GA* can protect against AlCl_3_ toxicity.

## Introduction

Aluminum (AL) is a major heavy metal that is widely distributed in the environment because of its use in several industries [1]. AL is the third most common metal and the most abundant element in the earth’s crust, accounting for approximately 8% of all mineral components [2]. AL is widely used in a variety of industries and items, including medicines, cosmetics, cans, cooking utensils, signs, building materials, aerospace industry, water purification, and metal alloy production [3]. Aluminum chloride (AlCl_3_) is a commonly used AL derivative. AlCl_3_ can accumulate in the brain, kidneys, liver, and all mammalian organs, greatly increasing the levels of cytokines that induce inflammation and may cause serious health problems in humans [4]. In rats, histopathological examination revealed acute catarrhal inflammation and congestion in blood vessels of the testes, liver, and kidney following exposure to AL [5].

*Delonix regia* is a leguminous flowering plant with colorful blossoms and attractive red-orange peacock flowers [6]. *D. regia* is a semi-deciduous tree with many branches that is widely planted in tropical regions, particularly in Saudi Arabia [7]. *D. regia* is a plant with numerous beneficial phytoconstituents, including flavonoids, alkaloids, saponins, sterols, sitosterol, lupeol, tannins, carotenoids, and phenolic acids. Its ethanolic extracts have antioxidant, hepatoprotective, and anti-inflammatory properties [7, 8].

*Gum Arabic* (*GA*), a water-soluble dietary fibrous polysaccharide polymer, is derived from the dried gummy exudate of Acacia Senegal trees [9]. *GA* is high in Ca^2+^, Mg^2+^, and K^+^. *GA* is used in the pharmaceutical, cosmetic, and food industries [10]. Experimentally, *GA* has been used in the Middle East and North Africa to treat various diseases, including renal, hepatic, cardiac, anemia, and diabetes mellitus, as well as improve digestive systems and appetite [11].

The use of natural compounds as protective agents appears to be a viable and promising strategy [12, 13]. Studies in both *in vitro* and *in vivo* investigations have shown that *D. regia* with *GA* has important biological properties that protect against toxicity and provide other medical benefits [8, 11]. However, the effect of *D. regia* extract combined with *GA* remains unclear, necessitating scientific proof for its use in modern medicinal applications.

Therefore, it was necessary to shed light on the investigation of hematological, biochemical, histological, and histochemical alterations in *D. regia* extracts combined with *GA* as possible anti-toxic compounds following AlCl_3_ exposure. The present study explored the hypothesis that *D. regia* extracts combined with *GA* have a protective role against AlCl_3_ toxicity.

## Materials and Methods

### Ethical approval

All experimental protocols were approved by the Biomedical Research Ethical Committee at King Khalid University (ECM#2024-2803).

### Study period and location

The study was conducted during May and June 2024 in the Histology and Cell Biology Laboratory, Department of Biology, College of Science, King Khalid University, Saudi Arabia.

### *D. regia* and *GA*

**Figure-1:**
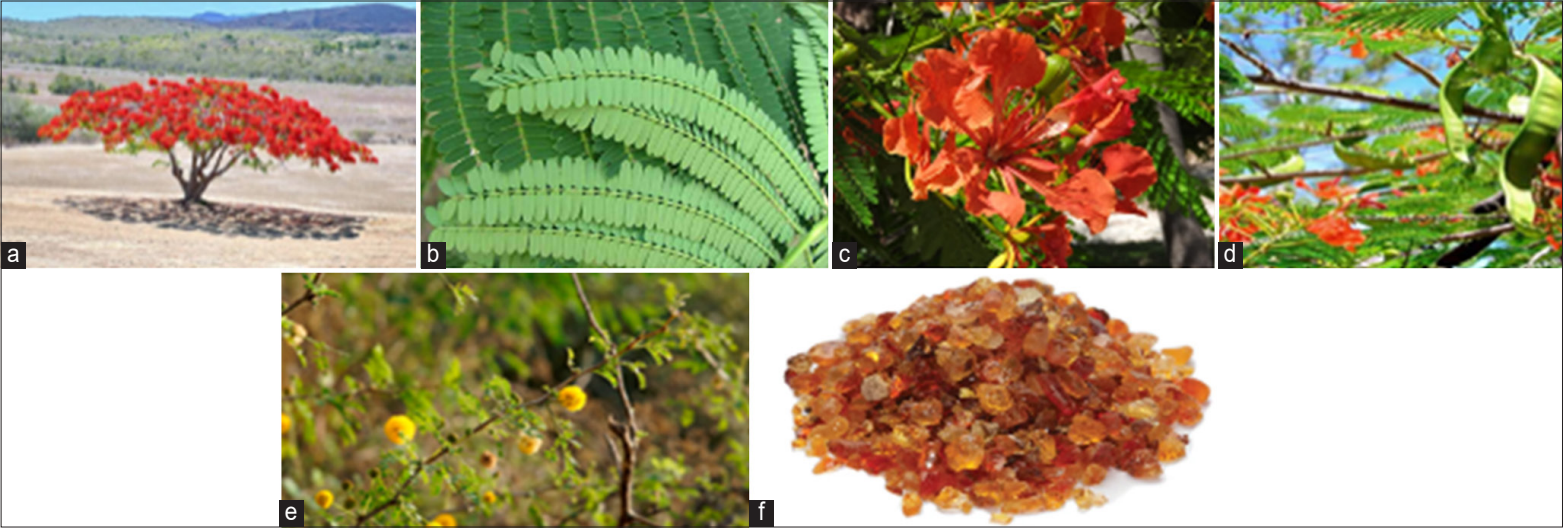
(a) Growth of *Delonix regia* trees. (b) *D. regia* leaves, (c) *D. regia* flowers, (d) *D. regia* young green legumes. (e) Acacia Senegal tree and (f) Gum Arabic.

During May and June, aerial components of *D. regia*, such as leaves, flowers, and young green (Figure-1), were collected from trees in Khamis Mushet, Asir, Saudi Arabia. The plant specimens were identified and confirmed by Mahmoud Fawzy, an expert from the Department of Biology, College of Science, King Khalid University, Abha, Saudi Arabia. Voucher specimens have been preserved in the herbarium of the Department of Biology at King Khalid University under voucher number #59792. The plant parts were crushed after drying in shade. A Soxhlet apparatus was used to extract 500 g of powder using methanol by Serag Eldin Elbehairi, an expert from the Tissue Culture and Cancer, Biology Department, College of Science, King Khalid University, Abha, Saudi Arabia. A rotary vacuum evaporator was used to dry the methanolic extract, yielding 100 g of brown residue [14]. *GA* was purchased from a local market (No. 0315000035; Al-Helal, Maka, Saudi Arabi). *GA* was dissolved in regular saline and administered orally once daily at a 15% w/v dosage (1 mL for each rat) [15].

### Experimental animals

Due to histological examination of all animals, only 20 Wistar male rats (*Rattus norvegicus*) were included in this study. Males were used in the study to avoid hormonal disturbances and physiological changes that may occur in the females. The animals, apparently healthy and clinically free of diseases, had a body weight of 185.79 ± 4.68 g and aged 115 ± 5 days, were included in this study. Rats were obtained from the Laboratory Animal Center, King Khalid University, Saudi Arabia. Animals were kept at a constant temperature (22 ± 2°C), humidity (55%), and light/dark conditions (12/12 h light/dark ratio). Animals were fed with commercial rat pellets, and drinking water was provided *ad libitum*. All animals were housed in polypropylene cages with chopped sawdust as bedding material. Animals were maintained under controlled environmental conditions. They were acclimatized for 1 week and randomly assigned to different groups.

### Experimental design

The animals were randomly assigned to four equal groups (5 rats/each): (i) CG served as the control group. (ii) AlCl_3_: Rats exposed to 80 mg/kg/body weight (BW) AlCl_3_ [16]. (iii) AlCl_3_ + *D. regia/GA*: Rats administered AlCl_3_ + 100 mg/kg B.W. with 15% B.W. of *D. regia* [17] and *GA* [18], respectively. (iv) *D. regia/GA*: Rats administered 100 mg/kg B.W. with 15% B.W. *D. regia* and *GA*, respectively. The experimental treatment was administered for 30 days.

### Biochemical evaluation

A total of 20 blood samples (1 sample × 4 groups × 5 animals) were collected after 30 days of AlCl_3_ exposure or *D. regia* and *GA* treatment using a capillary tube. The hematological parameters were evaluated using a hematology analyzer (SYSMEX XN-1000 SA-01, Sample Rack, Swiss) according to the method of Young and Donald [19]. Serum levels of aspartate aminotransferase (AST), alanine aminotransferase (ALT), alkaline phosphatase (ALP), urea, creatinine, uric acid, glucose cholesterol, and triglycerides were tested using a blood chemistry analyzer according to the diagnostic kit manufacturer instructions [20, 21]. Lipase profiles (lipase, amylase, triglyceride, cholesterol, high-density lipoprotein [HDL], and low-density lipoprotein [LDL]) were evaluated using a hematology analyzer (SYSMEX XN-1000 SA-01, Sample Rack, Swiss) according to Lee and David [22]. The electrolyte concentrations (Ca^++^, Na^+^, K^+^, Cl^-^, and Mg^++^) were evaluated as described by Nguyen-Khac *et al*. [23].

### Histopathology

After 30 days of experimentation, five animals from each group were euthanized to explore potential histological alterations. Small pieces of rat hepatic and renal tissues were excised and fixed in 10% formalin. Specimens were dehydrated with ethanol, cleared in xylene, and impregnated with molten paraffin wax in an oven at 60°C using an Automatic Linear Tissue Processor (ATP1000; Histo-line Laboratories, Italy). Tissues were embedded using a Leica EG1160 Tissue Embedding Center (Leica, Germany). Sections (4-5 μm) were cut and stained with hematoxylin/eosin and PAS using a staining machine (Multistainer Leica ST5020, Leica). The stained sections were inspected using an optical microscope (Olympus Microscope BP53 with Digital Camera, Japan), and all subsequent histopathological examinations were performed by an experienced pathologist who was unaware about the previous treatments.

### Statistical analysis

The data were analyzed using SPSS version 25 for Windows (IBM Corp., NY, USA). The Kolmogorov–Smirnov test confirmed that the data had a normal distribution. The biochemical parameter results are displayed as the mean ± standard deviation. p < 0.05 was deemed significant based on the statistical analysis, and differences in means across all groups were examined for significance using the one-way analysis of variance and Duncan’s test.

## Results

### Biochemical analysis

#### Hematological analysis

The hematological parameters, lymphocytes, and eosinophils showed significant abnormalities after AlCl_3_ exposure (Table-1). There was a significant (p < 0.05) increase in the white blood cells (WBCs) and platelets (PLT), while a significant (p < 0.05) decrease in the red blood cells (RBCs), hemoglobin (HGB), hematocrit, and mean corpuscular volume (MCV) after AlCl_3_ exposure compared with CG. Furthermore, the presented study showed that a significance (p < 0.05) improved for the hematological parameters in the AlCl_3_ +*D. regia/GA*-treated group. Furthermore, this study found a significant (p < 0.05) increase in lymphocytes, monocytes, eosinophils, and basophil levels after AlCl_3_ exposure compared with CG. Moreover, treatment with *D. regia/GA* during AlCl_3_ or alone significantly improved lymphocytes, monocytes, eosinophils, and basophils compared with the AlCl_3_ group (p < 0.05).

**Table-1:** The hematological parameters in the CG, AlCl_3_, AlCl_3_ + *D. regia/GA*, and *D. regia/GA* in Wistar male rats.

Hematological parameter	CG	AlCl_3_	AlCl_3_ + *D. regia/GA*	*D. regia/GA*	SEM	Significance
WBCs	6.24^b^	7.66^a^	6.2^b^	6.56^c^	0.85	0.05
RBCs	7.9^a^	7.63^b^	7.85^b^	7.72^c^	0.02	0.05
PLT	379.66^b^	549^a^	388^b^	368.6^b^	2.12	0.05
HGB	17.27^a^	14.60^c^	16.41^b^	16.87^bc^	0.98	0.05
HCT	53.40^a^	30.03^c^	51.10^b^	48.43^b^	1.05	0.05
MCV	64.33^a^	57.77^b^	65.77^a^	63.23^a^	0.05	0.05
MCH	21.47	21.53	20.80	21.57	0.09	NS
MCHC	32.57	35.57	34.27	33.53	0.21	NS
Lymphocytes	25.97^c^	54.70^a^	38.27^b^	23.27^d^	0.11	0.05
Monocytes	2.27^c^	5.53^a^	2.10^b^	1.97^b^	1.52	0.05
Eosinophils	2.57^b^	6.33^a^	2.07^c^	1.83^d^	1.50	0.05
Basophils	0.20^c^	1.73^b^	1.57^a^	0.30^b^	0.65	0.05

^a,b,c,d^=Duncan’s test. RBC=Red blood cells, WBCs=White blood cells, PLT=Platelets, HGB=Hemoglobin, HCT=Hematocrit, MCV=Mean corpuscular volume, MCH=Mean corpuscular hemoglobin, MCHC=Mean corpuscular hemoglobin concentration, *D. regia*=*Delonix regia*, SEM=Standard error of the mean,*GA*=*Gum Arabic*, AlCl_3_=Aluminum chloride

#### Liver and kidney functions

The liver and kidney functions of animals exposed to AlCl_3_ and treated with *D. regia/GA* are shown in Table-2. The liver functions of AST, ALT, and ALP of animals exposed to AlCl_3_ showed a significant (p < 0.05) increase compared to CG. There were significant improvements in the AlCl_3_ + *D. regia/GA* treated group compared to AlCl_3_. On the other hand, treatment with *D. regia/GA* extract alone had no significant effect on the activities of AST, ALT, and ALP compared with CG. Furthermore, the kidney functions of creatinine, urea, and uric acid of the animals exposed to AlCl_3_ were a significant (p < 0.05) increase compared with CG. Further, co-administration of *D. regia/GA* with AlCl_3_ exhibited an ameliorative effect by significantly decreasing (p < 0.05) the levels of creatinine, urea, and uric acid when exposed to AlCl_3_.

**Table-2:** The liver and kidney functions enzymes in the CG, AlCl_3_, AlCl_3_ + *D. regia/GA*, and *D. regia/GA* in Wistar male rats.

Parameter	CG	AlCl_3_	AlCl_3_ + *D. regia/GA*	*D. regia/GA*	SEM	Significance
ALP (g/dL)	232.97^b^	275.43^a^	187.39^c^	239.30^b^	5.74	0.05
ALT (g/dL)	69.10^c^	123.63^a^	90.87^b^	73.33^c^	4.51	0.05
AST (g/dL)	138.43^b^	193.77^a^	120.27^c^	115.67^c^	2.51	0.05
Creatinine (mg/dL)	0.24^b^	0.34^a^	0.23^b^	0.20^b^	0.02	0.05
Urea (mg/dL)	1.63^c^	2.33^a^	1.60^c^	1.80^b^	0.05	0.05
Uric acid (mg/dL)	1.66^d^	2.36^a^	1.46^c^	2.03^b^	0.06	0.05

^a,b,c,d^=duncan test. ALP=Alkaline phosphates, ALT=Alanine aminotransferase, AST=Aspartate amino transaminase, *D. regia*=*Delonix regia*, SEM=Standard error of the mean, *GA*=*Gum Arabic*, AlCl_3_=Aluminum chloride

#### Glucose and lipid profiles

The glucose and lipase profiles (lipase, amylase, triglyceride, cholesterol, HDL, and LDL) during exposure to AlCl_3_ and *D. regia/GA* are presented in Table-3. The data revealed that rats exposed to AlCl_3_ had significantly (p < 0.05) increased serum levels of glucose, lipase, amylase, triglyceride, cholesterol, HDL, and LDL. However, the rats treated with *D. regia/GA* during AlCl_3_ exposure showed no significant (p > 0.05) increase in lipase, cholesterol, HDL, and LDL compared with CG, while increased (p < 0.05) for glucose, amylase, and triglyceride. Moreover, treatment with *D. regia/GA* during AlCl_3_ exposure or alone significantly improved glucose, lipase, amylase, triglyceride, cholesterol, HDL, and LDL compared with AlCl_3_.

**Table-3:** Glucose and lipid profile of CG, AlCl_3_, AlCl_3_ + *D. regia/GA*, and *D. regia/GA* in Wistar male rats.

Parameter	CG	AlCl_3_	AlCl_3_ + *D. regia/GA*	*D. regia/GA*	SEM	Significance
Glucose (mg/dL)	83.59^c^	104.85^a^	98.97^b^	80.15^c^	0.52	0.05
Lipase (g/dL)	7.70^c^	11.53^a^	6.24^c^	10.33^b^	0.08	0.05
Amylase (g/dL)	3.40^c^	5.63^a^	4.57^b^	4.34^b^	0.01	0.05
Triglyceride (mg/dL)	117.06^b^	143.46^a^	112.06^d^	118.33^c^	1.01	0.05
Cholesterol (mg/dL)	70.63^b^	78.2^a^	60.86^b^	70.23^b^	0.09	0.05
HDL (g/dL)	61.12^c^	38.30^a^	42.81^c^	45.44^b^	2.02	0.05
LDL (g/dL)	11.03^c^	15.53^a^	9.93^c^	14.33^b^	0.06	0.05

^a,b,c,d^=Duncan’s test. HDL=High-density lipoprotein, LDL=Low-density lipoprotein, *D. regia*=*Delonix regia*, SEM=Standard error of the mean, *GA*=*Gum Arabic*, AlCl_3_=Aluminum chloride

#### Electrolytes concentration

The electrolyte concentrations (Ca, Na, K, Cl, and Mg) of the animals during exposure to AlCl_3_ and *D. regia/GA* are presented in Table-4. There were no significant (p > 0.05) differences in Ca and Na concentrations between the groups. However, rats exposed to AlCl_3_ had significantly (p < 0.05) elevated K, Cl, and Mg levels compared to the other groups. In contrast, treatment with *D. regia/GA* during or after AlCl_3_ exposure significantly improved K, Cl, and Mg levels compared with AlCl_3_.

**Table-4:** Electrolyte concentrations (Ca, Na, K, Cl, and Mg) in CG, AlCl_3_, AlCl_3_ + *D. regia/GA*, and *D. regia/GA* in Wistar male rats.

Electrolyte	CG	AlCl_3_	AlCl_3_ + *D. regia/GA*	*D. regia/GA*	SEM	Significance
Ca	10.9	12.19	13.41	12.133	0.09	NS
Na	143	147.33	143	148.33	0.04	NS
K	6.18^b^	8.55^a^	7.03^b^	7.05^b^	0.01	0.05
Cl	106.53^b^	120^a^	105.06^b^	107.6^b^	0.02	0.05
Mg	2.20^b^	3.30^a^	2.79^b^	2.33^b^	0.01	0.05

^a,b,c,d^=Duncan’s test. *D. regia*=*Delonix regia*, SEM=Standard error of the mean, *GA*=*Gum Arabic*, AlCl_3_=Aluminum chloride

### Histopathological examination

#### Histopathology of the liver

**Figure-2:**
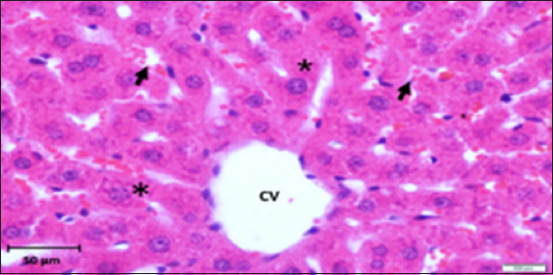
Photomicrograph of a section in the liver of a control rat showing a normal hepatic lobule with a central vein (cv). Note that hepatocytes are radiate from central vein as hepatic cords (*) separated from each other by blood sinusoids (arrows). Hematoxylin and eosin stain (400×).

**Figure-3:**
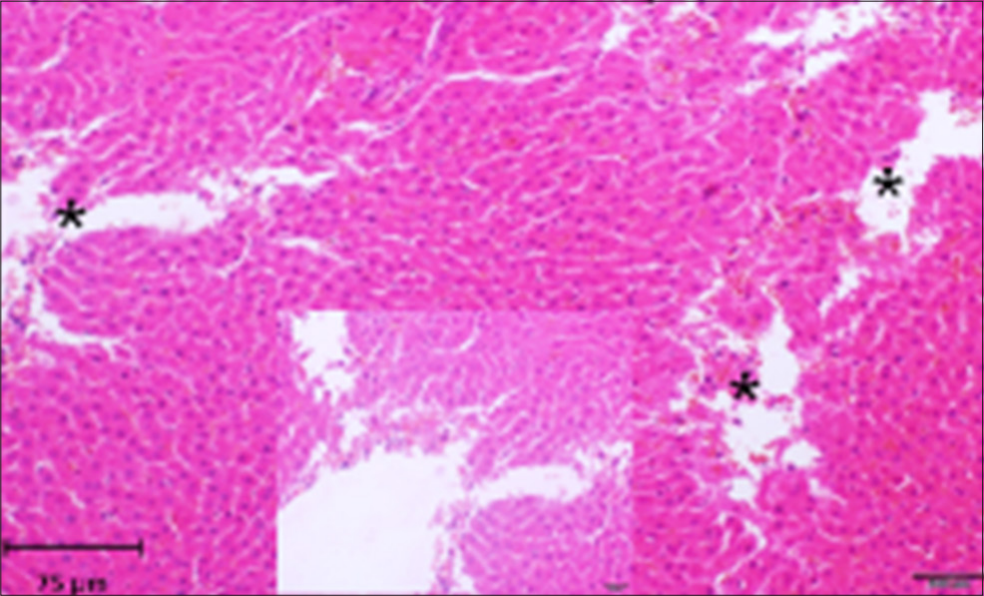
Photomicrograph of a section of the liver of rats exposed to AlCl_3_ showing necrotic foci (*). Hematoxylin and eosin stain (200×).

**Figure-4:**
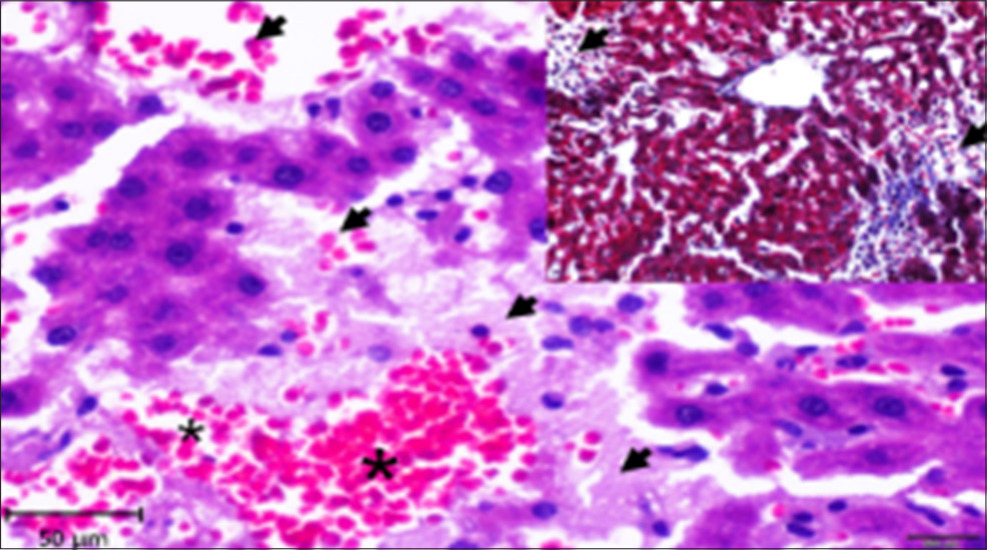
Photomicrograph of a section in the liver of rats exposed to AlCl_3_ showing hemorrhage foci (*) associated with necrotic foci (arrows) Hematoxylin and eosin stain (400×). Inset: Masson trichrome stain (200×).

**Figure-5:**
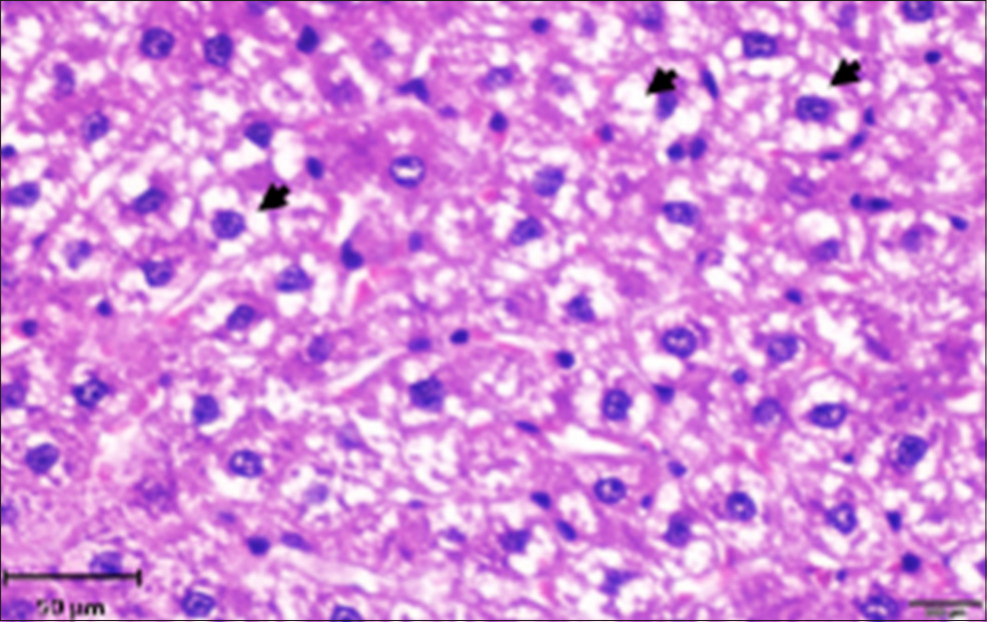
Photomicrograph of a section of the liver of rats exposed to AlCl_3_ showing ballooning and vacuolization degenerative changes in hepatocytes (arrow). Hematoxylin and eosin stain (400×).

**Figure-6:**
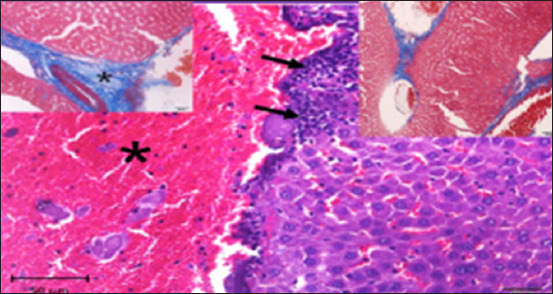
Photomicrograph of a section of the liver of rats exposed to AlCl_3_ showing perivascular infiltration of lymphocytes (arrow) around the dilation of congested blood vessels (*). Hematoxylin and eosin stain (400×). Inset: mild fibrosis (*) around the blood vessel. Masson trichrome stain (400 ×).

**Figure-7:**
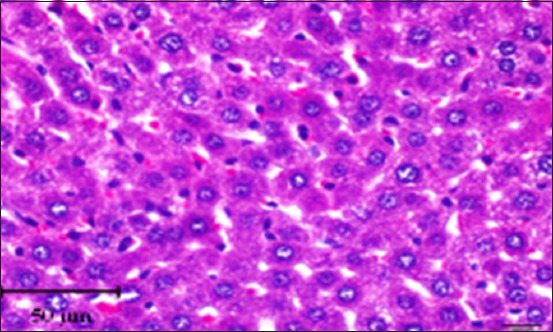
Photomicrograph of a section in the liver of *a Delonix regia/*gum Arabic rat showing recovery with strong reaction (magenta). PAS stain. (a 200 ×, b 400 ×).

Histopathology of the liver in the CG group showed a normal histological structure with central veins and normal nodules of all the components of the hepatic lobules and portal area; hepatocytes and their nuclei, Kupffer cells, blood sinusoids, and blood vessels were also normal with no indication of any abnormal histological changes (Figure-2). On the other hand, liver tissue sections in the AlCl_3_ showed degenerative foci with dispersed hemorrhagic foci, as well as dilatation of blood sinusoids, capillaries, and necrotic foci linked with hemorrhage (Figures-3–6). In addition, vacuolization and degenerative alterations in hepatocytes and perivascular lymphocyte infiltration around congested and dilated blood vessels were observed (Figures-3–6). Histological sections of the AlCl_3_ + *D. regia/GA* group showed reduced hepatocellular damage and inflammation and a normal liver architecture. The treatment reduced hepatic fibrosis and fat accumulation while dilating the central vein. This suggests that *D. regia/GA* might be an effective protective agent (Figure-7). Histological examination of *D. regia/GA* revealed a normal liver pattern.

#### Histopathology of kidney

**Figure-8:**
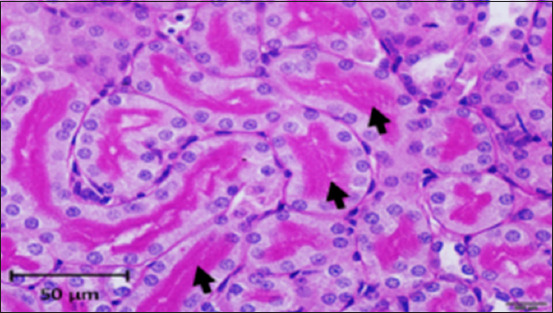
Photomicrograph of a section in the kidney of a control rat showing strong reaction (magenta), especially in the brush borders of the proximal convoluted tubules (arrows) and the basement membrane. PAS stain (400 ×).

**Figure-9:**
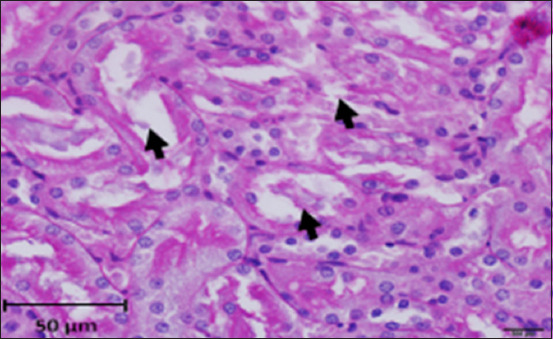
Photomicrograph of a section in the kidney of rats exposed to AlCl_3_ showing weak reaction (magenta) in brush borders of proximal convoluted tubules (arrows) and basal lamina. PAS stain (400×).

**Figure-10:**
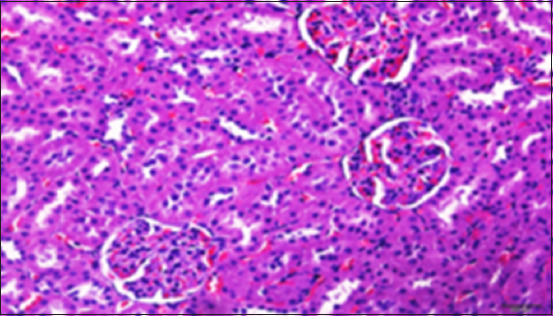
Photomicrograph of a section in the kidney of rats exposed to AlCl_3_ + *Delonix regia*/Gum Arabic, showing improved renal structure and few hydropic changes. Hematoxylin and eosin stain (400×).

**Figure-11:**
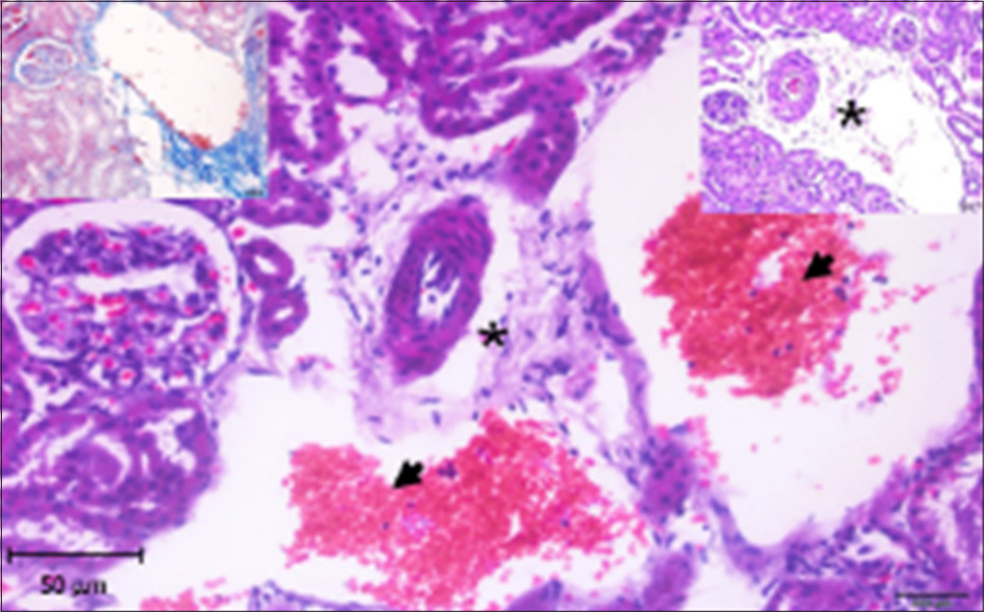
Photomicrograph of a section of the kidney from rats exposed to ALCl_3_ showing dilation of blood capillaries (arrowheads) and edematous material infiltration around the blood vessel (*). Hematoxylin and eosin stain (1000×). Inset left: Masson trichrome stain (200×).

**Figure-12:**
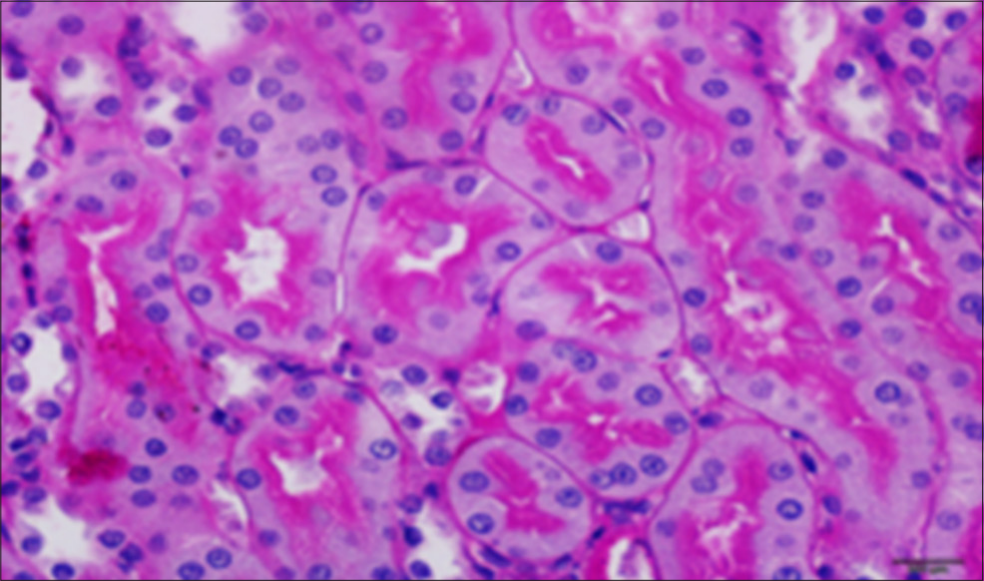
Photomicrograph of a section in the kidney of AlCl_3_ + *Delonix regia*/Gum Arabic rat showing recovery with strong reaction (magenta), especially in brush borders of the proximal convoluted tubules (arrows) and basement membrane. PAS stain (400×).

Histological analysis of the kidney tissue in the CG revealed normal renal tubules, corpuscles, glomeruli of all nephron components, and intratubular tissues, with no evidence of aberrant histological changes (Figure-8). A histological analysis following ALCL_3_ exposure revealed that the rat renal tissue had cortical dilatation and congestion of intertubular blood capillaries, as well as necrotic foci with degenerative alterations in certain tubules of the cortex. Vacillation and hydropic degenerative alterations followed by tubular vacuolization, eosinophilia material infiltration into the tubules, and tubular vacuolization in convoluted tubule cells. In addition, collagen fibers accumulate in the renal tissue, as do scatter hemorrhages with localized necrosis (Figures-9–12).

**Figure-13:**
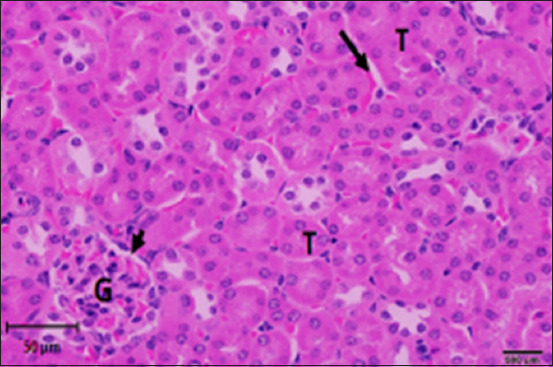
Photomicrograph of a section in the kidney of a Delonix regia/gum Arabic rat showing normal with strong reaction (magenta), especially in the brush borders of the proximal convoluted tubules (arrows) and the basement membrane. PAS stain (400×).

In this study, histological sections of the renal tissue from rats exposed to AlCl_3_ and treated with *D. regia/GA* revealed reduced tubular damage and interstitial inflammation and preserved renal tissue architecture. The architecture of renal tissue appears to have been preserved, with minor changes in glomerular size and Bowman’s capsule thickness. However, bleeding was observed in multiple areas of the renal parenchyma (Figure-13). All the members of this group possessed histological structures similar to those of *D. regia/GA*, with no alterations found in any of the organs examined compared with the CG (Figure-13).

## Discussion

This study revealed the harmful consequences of AlCl_3_-induced histological and biochemical changes. Furthermore, the current investigation found that *D. regia* extracts mixed with *GA* significantly enhanced recovery to a normal condition following AlCl_3_ exposure and improved liver and kidney function. However, *D. regia* extract mixed with *GA* improved liver and kidney histology characteristics.

Our study revealed that the hematological, biochemical, and histopathological markers of albino rats were affected following 30 days of exposure to 80 mg/kg body weight of AlCl_3_. There was a significant increase in lymphocytes, white blood cells, mean corpuscular HGB, MCV, and PLT in albino rats treated for 8 weeks with 40 mg/L AlCl_3_ [24]. Elevated WBC and lymphocyte count in exposed rats may be connected to an increased risk of developing organ diseases because of AlCl_3_ exposure. AL may alter erythropoiesis (RBC production) by affecting mature erythrocytes and cellular metabolism in late progenitor cells [25]. AL generates free radicals and reactive oxygen species in cells. Fatty acid superoxidase production and cellular membrane protein oxidation reduces cellular membrane fluidity and damage the membrane itself [26]. AlCl_3_ may accumulate in the liver, kidneys, brain, and other mammalian organs, significantly increasing the levels of cytokines that cause inflammation and potentially negatively impacting human health [17].

This study reported that treatment with *D. regia* extract with *GA* considerably improved liver integrity due to increased levels of critical liver enzymes and the ability to protect against liver damage, which might be ascribed to the plant extract’s antioxidant characteristics. Our findings on the hepatoprotective properties of *D. regia* extract with *GA* support the findings of Nwawuba *et al*. [7].

In the present study, AlCl_3_-treated rats had greater plasma magnesium, calcium, potassium, and sodium levels, which might constitute a concern in specific instances. In contrast, treatment with *D. regia/GA* improved these parameters. Giving *D. regia* *GA*, which contains calcium, potassium, and sodium, may safely increase the levels of these electrolytes in the plasma [27].

Our results demonstrated that oral treatment with AlCl_3_ substantially increased AST, ALT, and ALP. These results were consistent with the findings of Abdel Ghfar *et al*. [28] and Syaad *et al*. [29], who reported that oral AL therapy increased hepatic enzyme activity in rats following AlCl_3_ exposure. In addition, AL exposure causes alterations in hepatic membrane permeability [30]. However, this study found that *D. regia/GA* treatment substantially reduced ALT, AST, and ALP. Our findings are consistent with those of El-Gizawy *et al*. [31], who discovered that the ethyl acetate fraction of *D. regia* leaves has a robust and considerable hepatoprotective effect that may be linked to its high phenolic chemical content, indicating hepatocyte cell membrane stability.

In this study, rats exposed to AlCl_3_ had significantly higher creatinine, urea, and uric acid contents. Increased blood creatinine levels are typically thought to indicate abnormal renal function [13, 32]. However, our study showed that *D. regia/GA* considerably lowered lipid markers, such as total cholesterol and triglycerides, which is consistent with Al-Jubori *et al*. [11], who reported that *GA*, due to its high fiber content, can lower cholesterol levels.

In the present study, histopathological findings included necrosis, tissue degradation, sinusoidal dilatation, inflammatory cell infiltration, and blood vessel congestion in rats exposed to AlCl_3_. These findings are consistent with those of Benzaid *et al*. [33]. This study showed that *D. regia* extracts and *GA* improved renal and hepatic damage, indicating their preventive ability. These findings support the findings of Wang *et al*. [34], who concluded that *D. regia* extracts have protective qualities by activating vasodilation and regulating the tumor necrosis factor-alpha pathway. Shewale *et al*. [35] also reported that the ethanol extract of *D. regia* leaves displayed considerable anti-inflammatory activity at a dosage of 400 mg/kg when compared with a control group in several animals. These data indicate that *D. regia* extracts might be a potential herbal medicine for protection against AlCl_3_ toxicity.

## Conclusion

The current study indicated that exposure to AlCl_3_ for 30 days had negative effects on biochemical and histological indicators. This study also found that oral supplementation with 15% of *D. regia* extract with *GA* at 15% BW significantly enhanced recovery to a normal condition following AlCl_3_ exposure; moreover, these substances improved biochemical liver and kidney functions. Furthermore, the combination of *D. regia* extract and *GA* improved liver and kidney. The findings of this study permit the presentation of a therapeutic plan that uses *D. regia* extract and *GA* to reduce AlCl_3_ toxicity. Acquiring this insight will be the first step toward creating one-of-a-kind remedies that use natural antitoxins.

## Authors’ Contributions

AAA, MAA, AAS, and AMA: Conceptualized and designed the study, blood sampling, statistically analyzed the data, and drafted the manuscript. HSA, AMS, AMA, and ASA: Conducted the field study and designed the study. MHA, AAE, and MA: Performed blood sampling and data collection. MM and MEA: Performed interpretation of the results, statistically analyzed the data and drafted the manuscript. All authors have read and approved the final manuscript.
